# Retraction: Abdallah MS, Ramadan AN, Omara‐Reda H, et al. Double‐blind, randomized, placebo‐controlled pilot study of the phosphodiesterase‐3 inhibitor cilostazol as an adjunctive to antidepressants in patients with major depressive disorder. *CNS Neurosci. Ther.* 2021;27:1540–1548 (https://doi.org/10.1111/cns.13731)

**DOI:** 10.1111/cns.14667

**Published:** 2024-03-11

## Abstract

**Retraction:** Abdallah MS, Ramadan AN, Omara‐Reda H, et al. Double‐blind, randomized, placebo‐controlled pilot study of the phosphodiesterase‐3 inhibitor cilostazol as an adjunctive to antidepressants in patients with major depressive disorder. *CNS Neurosci. Ther.* 2021;27:1540–1548 (https://doi.org/10.1111/cns.13731)

The above article, published online on September 21, 2021 in Wiley Online Library (wileyonlinelibrary.com) has been retracted by agreement between the journal's Editor‐in‐Chief, Xiaoming Hu, and John Wiley and Sons Ltd.

Following publication, multiple concerns were raised by a third party about the integrity of the clinical trial. An investigation revealed significant issues regarding the reliability of the reported data. As a result, the Editor‐in‐Chief no longer has confidence in the validity of the results presented in this article.

**Retraction:** Abdallah MS, Ramadan AN, Omara‐Reda H, et al. Double‐blind, randomized, placebo‐controlled pilot study of the phosphodiesterase‐3 inhibitor cilostazol as an adjunctive to antidepressants in patients with major depressive disorder. *CNS Neurosci. Ther.* 2021;27:1540–1548 (https://doi.org/10.1111/cns.13731)

The above article, published online on September 21, 2021 in Wiley Online Library (wileyonlinelibrary.com) has been retracted by agreement between the journal's Editor‐in‐Chief, Xiaoming Hu, and John Wiley and Sons Ltd.

Following publication, multiple concerns were raised by a third party about the integrity of the clinical trial. An investigation revealed significant issues regarding the reliability of the reported data. As a result, the Editor‐in‐Chief no longer has confidence in the validity of the results presented in this article.

